# A voyage of discovering the impacts of teacher immunity and emotion regulation on professional identity, autonomy, and work motivation in Iranian EFL landscape

**DOI:** 10.1186/s40359-024-01544-9

**Published:** 2024-01-22

**Authors:** Ehsan Namaziandost, Tahereh Heydarnejad, Afsheen Rezai, Karamollah Javanmard

**Affiliations:** 1 Department of General Courses, Ahvaz Jundishapur University of Medical Sciences, Ahvaz, Iran; 2 Department of English Language Teaching, Ahvaz Branch, Islamic Azad University, Ahvaz, Iran; 3 Department of English Language, Faculty of Literature and Humanities, University of Gonabad, Gonabad, Iran; 4 Department of Teaching English and Linguistics, Faculty of Literature and Humanities, University of Ayatollah Ozma Borujerdi, Borujerd, Iran; 5 Department of Social Science, Ayatollah Ozma Borujerdi University, Borujerd, Iran

**Keywords:** Immunity, Emotion regulation, Professional identity, Teacher autonomy, Work motivation, EFL teachers

## Abstract

The literature has widely recognized the significance of examining the influence of teacher professional identity (TPI), teacher autonomy (TA), and teacher work motivation (TWM) on teacher well-being. However, there is a noticeable gap in the research regarding the role of teacher immunity (TI) and teacher emotion regulation (TER) in TPI, TA, TWM among English as a foreign language (EFL) teachers. In light of this gap, the present study aimed to investigate the association between TI and TER with TPI, TA, and TWM of EFL teachers. A sample of 433 EFL teachers completed the Language Teacher Immunity Instrument, the Language Teacher Emotion Regulation Inventory, the Teacher Professional Identity Scale, the Teacher Autonomy Questionnaire, and the Multidimensional Work Motivation Scale. The Confirmatory Factor Analysis and Structural Equation Modeling were conducted to analyze the data. Findings disclosed that TI and TER were strong predictor of TPI, TA, and TWM among the EFL teachers. The study concludes by offering some pedagogical implications for different stakeholders and discussing potential areas of research that can be developed based on the findings.

**Supplementary Information:**

The online version contains supplementary material available at 10.1186/s40359-024-01544-9.

## Introduction

English as a Foreign Language (EFL) education plays a crucial role in enhancing communication skills and promoting global understanding. Within this context, teachers have a significant impact on students’ language learning outcomes and overall educational experiences [1, 2]. On the other hand, the efficiency of EFL teachers to accomplish their job duties may be affected by different factors, such as teacher immunity (TI) [3], teacher emotion regulation (TER) [4], teacher professional identity (TPI) [5], teacher autonomy (TA) [6], and work motivation [7].

In recent years, TI has gained attention due to its critical role in the well-being and effectiveness of teachers [8]. According to [9], TI refers to the ability of teachers to withstand and recover from emotional stressors and demands of their profession. It involves a combination of psychological, physical, and emotional well-being that enables teachers to cope with the challenges they face in their teaching careers [9]. Understanding and developing TI is essential as it contributes to overall teacher well-being and job satisfaction, which in turn can positively impact students’ learning experiences and outcomes [10]. TER is another important aspect that influences teacher performance and well-being [11]. As defined by [12], it refers to the process of managing and modulating one’s emotions in order to adaptively respond to various situations. Effective TER allows teachers to navigate the complexities of their profession, handle stressful situations, and maintain positive student-teacher relationships [13]. By regulating their emotions, teachers can create a supportive and conducive learning environment, enhance their own job satisfaction, and ultimately improve their teaching effectiveness [14].

Another construct explored in this study is TPI which refers to the beliefs, values, and attitudes that teachers associate with their role as educators [15, 16]. It is a crucial component that influences teachers’ behaviors, instructional practices, and overall job satisfaction [17–19]. TI and TER can significantly affect the development and maintenance of TPI. When teachers have high levels of TI and effective TER strategies, they are more likely to experience a stronger sense of TPI, leading to increased job satisfaction and improved teaching practices [8, 20]. TA, as another construct, refers to the level of independence and decision-making power that teachers have in their professional roles [21]. It is a crucial factor in promoting teacher motivation, job satisfaction, and overall well-being [22]. TI and TER can influence TA by enabling teachers to better navigate challenges, manage their emotions, and advocate for their professional needs in the classroom and educational contexts [23]. As noted by [22], a balance between TA and effective TER can lead to a more fulfilling teaching experience and enhance overall job satisfaction. The last construct in this study is teacher work motivation which is the internal drive and desire to engage in professional tasks and achieve desired goals [24]. It plays a vital role in ensuring teachers’ dedication, persistence, and effectiveness in their profession [25]. TI and TER can have an impact on work motivation. When teachers possess a high level of TI and effective TER strategies, they are more likely to experience higher levels of work motivation, leading to increased productivity, job satisfaction, and improved teaching practices [13, 26].

Given the points discussed above, it is essential to explore the association between TI and TER with TPI, TA, and work motivation among EFL teachers in Iran. To address this, the present study aimed to disclose the role of TI and TER in TPI, TA, and work motivation among EFL teachers in Iran. The significance of this study lies in the fact that it addresses a gap in the existing literature on the relationship between TI, TER, TPI, TA, and work motivation in the context of EFL teaching in Iran. While there is some research on these topics in other contexts, there is a lack of studies that specifically focus on the EFL context in Iran. By investigating the impact of TI and TER on TPI, TA, and work motivation, this study has the potential to contribute to a better understanding of the factors that influence the job satisfaction and performance of EFL teachers in Iran. This, in turn, can inform the development of policies and practices that support the well-being and professional development of EFL teachers in Iran and other similar contexts. Furthermore, this study may also have broader implications for the field of education and psychology, as it sheds light on the complex interplay between individual factors (e.g., TI and TER) and contextual factors (e.g., TPI, TA, and work motivation) in shaping job satisfaction and performance. To meet these purposes, the following research questions were investigated:

- RQ1. Are TI and ER significantly associated with TPI among Iranian private language school EFL teachers?
- RQ2. Are TI and ER significantly associated with TA among Iranian private language school EFL teachers?
- RQ3. Are TI and ER significantly associated with WM among Iranian private language school EFL teachers?

## Literature review

### Teacher immunity

The concept of immunity originates from the Latin word Immunis, meaning resistance or exemption from something. It refers to the defense mechanism that protects against harmful, undesirable, or damaging effects from the external environment [9]. In biology, immunity is a defensive mechanism that activates naturally produced antibodies and prevents infection through biochemical responses [27]. Based on [28], TI is a protective and adaptive strategy that educators use to cope with various instructional challenges. TI involves a balance of crucial factors, such as the educator’s desire to teach, psychological well-being, and adaptability, as well as instructional demands, burnout, and disengagement [9] identified two specific aspects of language TI.

TI works like biological immunity, providing protection and defense in times of crisis. It helps educators improve their teaching effectiveness. Moreover, TI helps teachers develop their TPI, which can help them face future challenges [9]. also stressed the role of an individual’s identity, cognition, and behavior in social contexts in shaping and activating the immune response in educators. They claim that TI is often neglected in the study of language teacher motivation and identity. Identity is a complex construct that includes an individual’s past, present, and future experiences and how they make sense of them [29]. An individual’s identity can affect their level of effort, confidence, and mental well-being as a teacher. Additionally, identity is influenced by a mix of past, present, and expected experiences, as well as the personal meanings that individuals give to their situations [30].

Language TI can show up in two different ways: productive (positive) or maladaptive (negative). The maladaptive TI is similar to its biological counterpart. It can cause apathy, conservatism, cynicism, or resistance to change, which can hamper the teacher’s professional growth [9]. On the other hand, the productive TI, which is the positive form, can foster hope, commitment, enthusiasm, resilience, and motivation. The concept of TI emerged from an adaptation of complexity theory, especially self-organization theory [31]. Self-organization is the process by which the overall behavior of a dynamic system changes because of the interactions among its various components [31].

The literature has investigated the nature and development of TI among teachers in various contexts. For example [28] and [32], both examined the key aspects and types of TI, but they differed in the setting and the method. While [28] conducted a qualitative study on the self-image and motivation persistence of teachers in different settings and industries [32], used a mixed-methods approach to explore the common types and influencing factors of TI among Iranian English teachers. Similarly [33] and [28], both explored the predictors and outcomes of TI, but they differed in the model and the focus. While [33] created a model that predicts TI based on reflective teaching and TER [28], identified several key aspects of TI such as burnout, attrition, adaptability, teaching effectiveness, and the desire to teach. However, the literature does not provide enough research on TI in relation to language teaching effectiveness. Therefore, this study aims to address this gap.

### Teacher emotion regulation

Emotions are essential for teachers, especially language teachers. They affect various aspects of their professional lives, such as their social interactions [12], identities [16], self-efficacy [34], pedagogical practices [35], work engagement [13], self-regulation, and teaching style in higher education [36, 4] stress the centrality of emotions in the teaching profession.

The theories of appraisal and attribution help explain the emotions of educators [12]. The appraisal theory includes several sub-sections: goal consistency, goal conduciveness, coping potential, goal attainment/impediment responsibility, and goal significance [37]. It suggests a negative relationship between an individual’s emotional state and their situation. Attribution is a detailed analysis of the apparent causes of events [37]. Individuals use physiological, behavioral, and cognitive processes to regulate, evaluate, and control their emotions. This is called emotional regulation (ER) [38]. ER guides the emotions of individuals in different contexts [39, 40]. ER strategies help educators cope with both positive and negative emotions [13]. ER techniques have three basic elements: activating a supervisory goal, engaging regulatory processes, and modulating the emotional trajectory [35].

ER is the process of influencing one’s emotions, either by modifying their intensity, duration, or expression [41]. ER can be achieved through two main pathways: direct and indirect. Direct ER involves conscious and deliberate efforts to change one’s emotional state, such as reappraisal or suppression. Indirect ER involves less conscious and more automatic processes that influence one’s emotions indirectly, such as situation selection, situation modification, attention deployment, or seeking social support [42]. These processes form a continuum of ER strategies that vary in their degree of explicitness, control, and effort [42].

Based on the existing literature on ER [14], proposed a model for ER among language teachers. This model consists of six components: situation selection (SS), situation modification (SM), attention deployment (AD), reappraisal, suppression, and seeking social support (SSS). The first three components are derived from Gross’s process-based model of ER [39], which describes how people can regulate their emotions at different stages of the emotion-generative process. The fourth and fifth components are derived from Gross and John’s work on emotion regulation strategies [43], which distinguishes between cognitive reappraisal (changing the meaning of a situation) and expressive suppression (inhibiting the outward expression of emotions). The sixth component is derived from Taxer and Gross’s work on social aspects of ER [44], which emphasizes the role of seeking social support from others in coping with emotional challenges.

The literature has demonstrated the benefits of TER on teaching outcomes. For example [11, 13, 45], and [8] all found that TER was positively related to self-efficacy, reflective practice, and work engagement, but they differed in the context and the other variables involved. While [11] and [45] studied the students’ and teachers’ perspectives on TER and regulation strategies [13] and [8], examined the role of TER and ER on job satisfaction and burnout. Similarly [46] and [33], both found that TER was positively associated with resilience and TI, but they differed in the setting and the population. While [46] focused on Chinese EFL teachers, [33] investigated Iranian EFL teachers.

### Teacher professional identity

TPI is a dynamic and multifaceted construct that reflects how teachers perceive themselves and their roles in the educational context [47]. Different researchers have conceptualized TPI in different ways, but a common theme is that TPI involves a sense of self-awareness and self-involvement in teaching as a profession [5]. TPI develops through a process of identity formation and negotiation, in which teachers construct and connect different aspects of their professional selves. This process requires teachers to engage in self-reflection and self-regulation, as well as to balance the demands and challenges of their work environment [48]. TPI is also influenced by the social interactions and personal histories of teachers, as they shape their identity in relation to others and their own experiences [49]. TPI involves making conscious choices about one’s career path, professional affiliation, and personal competencies [50]. TPI is characterized by individual differences in traits, perspectives, beliefs, values, motivations, experiences, and relationships that define one’s professional roles and responsibilities [51]. The quality of a teacher’s TPI may affect their resilience, awareness, teaching skills, and intellectual abilities [52].

One of the models aims to illuminate TPI belongs to [53]. It is based on the idea that TPI is influenced by their emotional experiences and their emotional regulation strategies. The model proposes that teachers’ emotional experiences can be categorized into four types: positive, negative, mixed, and neutral. These emotional experiences can affect TPI in different ways, depending on how teachers regulate their emotions. The model suggests that teachers can use four types of ER strategies: reappraisal, suppression, expression, and avoidance [53]. These strategies can have different impacts on TPI development, such as enhancing, hindering, or maintaining it. The model also considers the contextual factors that may shape teachers’ emotional experiences and ER strategies, such as the teaching environment, the curriculum, the students, the colleagues, and the school culture [53].

The literature has explored the perceptions and development of TPI among teachers in various settings. For example, [54] and [55] both examined how TPI is related to teachers’ decisions to leave or stay in the profession, but they differed in the factors that influenced TPI. While [54] focused on the role of teachers’ value, efficacy, commitment, and emotions [55], considered the impact of personal experience, professional context, and external political environment. Similarly, [56] and [55] both investigated how TPI is mediated and changed over time, but they differed in the context and approach. While [56] studied the effect of a critical EFL teacher education course on Iranian teachers’ TPI reconstruction, [55] conducted a three-year study on the formation and mediation of TPI for secondary school teachers. However, the literature does not examine how TI and TER are associated with TPI in EFL contexts. Therefore, this study aims to investigate this issue among Iranian EFL learners.

### Teacher autonomy

Autonomy is a complex and contested concept that has different meanings and implications in various philosophical, social, and educational contexts [57]. Autonomy is not a fixed or innate trait that individuals have from birth; rather, it is a skill that can be learned and developed, depending on one’s abilities and circumstances [58]. Autonomy is especially important in education, as it relates to the roles and responsibilities of both teachers and learners [59]. According to [60], TA is a term that has been used in different ways to describe the degree of independence and self-regulation that teachers have in their work, particularly in the field of L2 instruction. TA can also refer to the ability and motivation of teachers to promote learner autonomy in their students [6]. TA emerged as a concept that linked the autonomy of teachers with the autonomy of learners, suggesting that teachers can facilitate learner autonomy by exercising their own autonomy in their teaching practices [6]. However, most of the research on TA has focused on the aspect of TA that relates to their professional development and career satisfaction [59, 61].

The literature has examined the effects of various psychological factors on TA and self-efficacy among teachers in different settings. For instance [58] and [8], both found that TA and self-efficacy were positively related to engagement, job satisfaction, and emotional exhaustion, but they differed in the role of other constructs such as productive immunity, ER, resilience, autonomy, and psychological well-being. While [58] focused on Norwegian teachers in elementary and middle schools [8], studied Iranian EFL university professors. However, the literature does not address how TI and TER are linked to TA and self-efficacy in EFL contexts. Therefore, this study aims to explore this issue among Iranian EFL learners.

### Teacher work motivation

Motivation is a key factor that affects one’s behavior and mental well-being. However, motivation is not a simple or clear-cut concept; rather, it is a complex and elusive phenomenon that has been defined in various ways by different scholars [7]. For example, [62] defines motivation as the process that involves “the initiation, direction, intensity, and persistence of behavior, particularly behavior aimed at attaining goals” (p. 3) [7]. also view motivation as an important construct that explains why people choose to engage in certain activities, how long they persist, and how much effort they invest. The level of motivation that teachers display in the classroom is directly related to their teaching efficacy, which refers to their teaching style, practice, behavior, and methods [63]. In a sense, teaching efficacy is influenced by various motivational factors, such as self-confidence, self-regulation, and goal orientation [64].

Self-determination theory (SDT) by [65] explains human motivation and how people make decisions about their actions. The theory is based on three key components: autonomy, competence, and relatedness. These components interact to influence an individual’s behavior, well-being, and performance, which can be applied to explain EFL teacher work motivation. The first component of SDT which refers to the need to be self-directed, taking initiative, and being in control of one’s own actions [66]. Teachers who have more control over the materials they teach, their instruction style, and the classroom environment will have more autonomous motivation. In contrast, teachers who feel micromanaged or controlled by external factors will feel less autonomous and may have low motivation at work [67]. The second component of SDT is competence which involves the desire to feel competent and effective in a particular activity [66]. Teachers who perceive they have a high level of competence in their EFL teaching performance will tend to have more motivation for work. Teachers who feel unsure or lack confidence in their abilities may not have much intrinsic motivation towards work. The third component of SDT is relatedness which focuses on the need for meaningful social connections with others in the workplace [65]. Teachers who have positive relationships or feel connected to their colleagues or students will have higher motivation and job satisfaction [67]. On the other hand, teachers who isolate or experience negative interactions with others may have low motivation at work. Overall, self-determination theory can be used to explain EFL teacher work motivation since it takes into account extrinsic and intrinsic factors that can impact teacher motivation. Teachers who feel autonomous, competent, and related to others in the workplace are more likely to be motivated and engaged in their work. On the other hand, teachers who feel controlled, incompetent, and disconnected from others may have low motivation and job satisfaction.

Several studies have investigated the factors that affect TI among EFL teachers in different contexts. For example, [68] and [69] both found a positive relationship between TI and motivation, but they differed in the role of psychological well-being and work engagement. While [68] reported that teachers in private institutes had higher levels of these variables than teachers in public schools in Iran [69], showed that psychological well-being was a better predictor of TI than work engagement across four Asian countries (China, Iran, Pakistan, and Turkey). However, the literature lacks research on how TI and TER are related to TWM in EFL settings. Therefore, this study aims to fill this gap.

## Method

### Settings and participants

The present investigation comprised a cohort of 433 Iranian EFL teachers who were engaged in teaching English as a foreign language in Mashhad, Iran. The participants were selected from 52 private language institutes and constituted a population of 2470 EFL teachers. Of the total sample, two hundred and one were male and two hundred and thirty-two were female. The selection of participants was carried out through the use of a random sampling technique, which guarantees that each member of the population has an equal probability of being chosen [70]. This approach facilitates the attainment of a representative sample that can yield valid and reliable outcomes. To ensure the external validity of this study, careful consideration was given to the selection of participants based on a range of demographic variables such as age, gender, years of teaching experience, and work locations. The age range of the participants was between 22 and 49, and their years of teaching experience varied from 1 to 27 years. The participants held degrees in English Teaching (*n* = 151), English Literature (*n* = 98), English Translation (*n* = 132), and Linguistics (*n* = 52). Furthermore, their educational qualifications varied, and they held either Ph.D. (*n* = 42), M.A. (*n* = 294), or B.A. (*n* = 107) degrees. It is noteworthy that the study protocol was approved by the Research Ethics Committee of Gonabad University (NO: 37/298716-219), and the participants provided written informed consent to partake in this study.

### Instruments

#### Language teacher immunity instrument (LTII)

The present study assessed the teachers’ level of immunity using the LTII, which was developed and validated by [28]. The LTII comprises 39 items that are rated on a five-point Likert scale ranging from 1 (strongly disagree) to 5 (strongly agree). This instrument measures several dimensions related to teachers’ psychological well-being, including self-efficacy (7 items) (e.g., “I feel I am positively influencing my students’ lives through my teaching.”), burnout (5 items) e.g., “There are days at school when I feel vulnerable.”), resilience (5 items) (e.g., “Failures double my motivation to succeed as a teacher.”), attitudes toward teaching (5 items) (e.g., “Teaching is my life and I can’t imagine giving it up.”), openness to change (6 items) (e.g., “I get frustrated when my work is unfamiliar and outside my comfort zone as a teacher.”), classroom affectivity (6 items) (e.g., “Overall, I expect more good things to happen to me in the classroom than bad.”), and coping (6 items) (e.g., “When things get really stressful, I try to come up with a strategy about what to do.”).

### **The language teacher emotion regulation inventory (LTERI)**

To measure the participants’ TER, the researchers utilized the LTERI, which was developed and validated by [14]. The LTERI was specifically designed to evaluate the ER techniques that are employed by EFL teachers. The participants were requested to reflect on their past experiences in the classroom before responding to the statements provided in the questionnaire. They were then asked to indicate which TER strategies they had employed during those experiences. The LTERI includes six sub-factors, such as situation selection (SS) (5 items) (e.g., “I avoid conflicting or emotionally disturbing situations in the staff room.”), situation modification (SM) (5 items) (e.g., “When an unpleasant discussion is raised in my classes, I try to change the topic.”), attention deployment (AD) (4 items) (e.g., “If I feel frustrated in language classes, I try to engage myself in different class activities to forget it.”), reappraisal (5 items) (e.g., “If my students’ misbehavior makes me angry, I remind myself that they are inexperienced.”), suppression (4 items) (e.g., “If I feel anxious in my language classes, I try to suppress that.”), and seeking social support (SSS) (4 items) (e.g., “If I feel nervous in my language classes, I talk about it with someone who can understand me.”). The LTERI consists of 27 items that are scored on a five-point Likert scale, ranging from 1 (never) to 5 (always).

### Teacher professional identity scale (TPIS)

In order to assess the participants’ TPI, the Teacher Professional Identity Scale (TPIS) was utilized. This instrument, which was designed and validated by [71], consists of 22 items that were rated on a 5-point Likert scale ranging from 1 (strongly disagree) to 5 (strongly agree). The six sub-scales of TPIS contain self-expectation (5 items) (e.g., “I hope I can continue to teach for the rest of my life.”), duties of teachers (3 items) (e.g., “I believe that being responsible for students is one of my professional duties.”), external influences (4 items) (e.g., “School policies influence my teaching.”), pedagogy (3 items) (e.g., “As a teacher, I always lead by example to teach students how to get along with others.”), instruction skills and knowledge (4 items) (e.g., “I believe that teachers should be able to use appropriate teaching methods to stimulate students’ interest in learning”.), and teachers’ citizenship behavior (4 items) (e.g., “I commit myself extensively to my job as a teacher.”).

### Teacher autonomy questionnaire (TAQ)

To evaluate the participants’ level of TA, the Teacher Autonomy Questionnaire (TAQ) was utilized [57]. developed and validated this instrument specifically for the purpose of measuring the autonomy of language teachers. TAQ gauges two sub-factors, namely general autonomy (12 items) (e.g., I am free to be creative in my teaching approach.”) and curriculum autonomy (11 items) (e.g., “The evaluation and assessment activities are selected by others.”). The TAQ makes use of a 5-point Likert scale, ranging from 1 (= strongly disagree) to 5 (= strongly agree).

### The multidimensional work motivation scale (MWMS)

To assess the participants’ level of work motivation (WM), the researchers employed the Multidimensional Work Motivation Scale (MWMS). This instrument, which was developed and validated by [72], comprises 19 items that are based on the self-determination theory. The MWMS measures six distinct factors related to work motivation, including amotivation (3 items) (e.g., “I do little because I don’t think this work is worth putting efforts into.”), extrinsic material regulation (EMR; 3 items) (e.g., “Because others will reward me financially only if I put enough effort in my job (e.g., employer, supervisor. . .”), extrinsic social regulation (ESR; 3 items) (e.g., “To avoid being criticized by others (e.g., supervisor, colleagues, family, clients. . .”), introjected regulation (InR4; items) (e.g., “Because I have to prove to myself that I can.”), identified regulation (IdR; 3 items) (e.g., “Because putting efforts in this job aligns with my personal values.”), and intrinsic motivation (IM, 3 items) (e.g., “Because what I do in my work is exciting.”). Answers were given via a 7-point Likert-scale ranging from 1 (= strongly agree) to 7 (= strongly disagree).

It is worth noting that prior to the study, the authors ensured the validity and reliability of the data collection tools. Two professors of Applied Linguistics evaluated the validity of the tools in terms of face and content, confirming their suitability for the study. To assess reliability, the authors administered the tools to a sample of 30 participants similar to those in the main study. The results of internal consistency measured through Cronbach Alpha were favorable, with scores of 0.94 for LTII, 0.82 for LTERI, 0.84 for TPIS, 0.78 for TAQ, and 0.89 for MWMS.

### Data collection and analysis procedures

Data collection for this study was conducted between June 2022 and September 2022, utilizing a web-based platform. The researchers employed Google Forms to administer an electronic survey to the participants, which included the LTII, LTERI, TPIS, TAQ, and MWMS. The web-based platform of the questionnaire allowed the authors to collect the required information from the participants without the time and location constraints. To prevent duplicate responses, the researchers enabled the Google Forms’ built-in ‘limit to 1 response’ option, which required the respondents to sign in with their Google account before filling up the form. The response rate was 82.3%, with a total of 581 completed surveys. However, 148 responses were eliminated because they were either incomplete, invalid, or outliers, resulting in a final sample size of 433 participants.

The data collected in this study was analyzed by performing a structural equation modeling (SEM) analysis using LISREL 8.80. This multivariate statistical technique is widely used to evaluate structural theories and is deemed to be highly reliable [73]. The SEM model is comprised of two components: the measurement model and the structural model. The measurement model is utilized to examine the correlation between observed and unobserved (latent) variables [74], while the structural model elucidates how the latent variables are interconnected.

## Results

This section provides an outline of the procedures involved in data screening. The results of descriptive statistics revealed that the LTII sub-factor, teaching self-efficacy, had the highest mean value (M = 23.66, SD = 7.24), while the LTERI component, Reappraisal, had the highest mean score (M = 17.12, SD = 4.54). Similarly, the TPIS sub-factor, Self-expectation, had the highest score (M = 17.04, SD = 4.62). Additionally, EFL teachers displayed the highest mean score for Attitude toward General Autonomy (M = 42.98, SD = 10.94) among TAQ components, while the leading MWMS component was Introjected regulation (M = 16.09, SD = 3.25).

The results of the Kolmogorov-Smirnov test showed that the data exhibits a normal distribution, which allows for the use of parametric tests. To examine the relationships between TI, TER, PI, TA, and TWM, both CFA and SEM were conducted using LISREL 8.80. To evaluate the adequacy of the model, four indices were employed, namely the Chi-Square/df ratio, Chi-Square, RMSEA, and NFI, GFI, and CFI [75]. recommends that the optimal values for these indices are a Chi-Square/df ratio less than three, a non-significant Chi-Square, an RMSEA less than 0.1, and NFI, GFI, and CFI values over 0.90.

In the subsequent stage, a Pearson product-moment correlation analysis was employed to investigate the correlation among the subscales of TI, TER, TPI, TA, and TWM. The results are informed in Tables 1 and 2.

**Table 1:** The correlation coefficients between TI, TER, TPI, TA, and TWM

	LTI	LTER	TPI	TA	MWM
LTI	1.000				
LTER	0.608^**^	1.000			
TPI	0.912^**^	0.504^**^	1.000		
TA	0.843^**^	0.542^**^	0.635^**^	1.000	
TWM	0.752^**^	0.628^**^	0.673^**^	^**^0.641	1.000

*Note*: LTI (Language Teacher Immunity); LTER (Language Teacher Emotion Regulation); TPI (Teacher Professional Identity); TA (Teacher Autonomy); MWM (Multidimensional Work Motivation)

Correlation is significant at the 0.01 level (2 tailed)**

Table 1 illustrates the significant associations that were found between TI, TER, TPI, TA, and TWM. Specifically, there was a correlation between TI, TPI (*r* = 0.912), TA (*r* = 0.843), as well as TWM (*r* = 0.752). Additionally, there was a link between TER, TPI (*r* = 0.504), TA (*r* = 0.542), as well as TWM (*r* = 0.628).

**Table 2:** The correlation coefficients between TI, TER, TPI, TA, and TWM subfactors

	LTI	LTER	Self-Expectation	Teachers’ Duties	External Influential Factors	Pedagogy	Instructional Skills and Knowledg	Teachers’ Citizenship Behavior	General Autonomy	Curriculum Autonomy	Amotivation	Extrinsic Material regulation	Extrinsic social regulation	Introjected regulation	Identified regulation	
LTI	1.000															
LTER	0.608^**^	1.000														
Self-Expectation	0.981^**^	0.558^**^	1.000													
Teachers’ Duties	0.935^**^	0.492^**^	0.684^**^	1.000												
External Influential Factors	0.905**	0.472**	0.558**	0.534**	1.000											
Pedagogy	0.896**	0.452**	0.604**	0.576**	0.678**	1.000										
Instructional Skills and Knowledge	0.971**	0.435**	0.672**	0.613**	0.544**	0.609^**^	1.000									
Teachers’ Citizenship Behavior	0.869^**^	0.435**	0.598**	0.624**	0.654**	0.598^**^	0.631^**^	1.000								
General Autonomy	0.823^**^	0.545**	0.703**	0.839**	0.741**	0.653^**^	0.724^**^	0.741^**^	1.000							
Curriculum Autonomy	0.818^**^	0.567**	0.784**	0.757**	0.732**	0.731^**^	0.812^**^	0.852^**^	0.559^**^	1.000						
Amotivation	-0.684^**^	-0.584**	-0.802**	-0.748**	-0.689**	-0.792^**^	-0.725^**^	-0.741^**^	-0.756^**^	-0.812^**^	1.000					
Extrinsic Material regulation	0.793^**^	0.682^**^	0.762^**^	0.633^**^	0.655^**^	0.803^**^	0.654^**^	0.809^**^	0.749^**^	0.789^**^	-0.557^**^	1.000				
Extrinsic social regulation	0.765^**^	0.657^**^	0.669^**^	0.689^**^	0.613^**^	0.656^**^	0.842^**^	0.859^**^	0.825^**^	0.751^**^	-0.648^**^	0.558^**^	1.000			
Introjected regulation	0.752^**^	0.649^**^	0.636^**^	0.831^**^	0.742^**^	0.631^**^	0.814^**^	0.644^**^	0.812^**^	0.688^**^	-0.698^**^	0.613^**^	0.579^**^	1.000		
Identified regulation	0.742^**^	0.664^**^	0.756^**^	0.754^**^	0.753^**^	0.882^**^	0.726^**^	0.731^**^	0.797^**^	0.725^**^	-0.553^**^	0.678^**^	0.642^**^	0.694^**^	1.000	
Intrinsic motivation	0.723^**^	0.608^**^	0.804^**^	0.723^**^	0.714^**^	0.832^**^	0.708^**^	0.645^**^	0.768^**^	0.744^**^	-0.574^**^	0.614^**^	0.633^**^	0.651^**^	0.578^**^	1.000

*Note*: LTI (Language Teacher Immunity); LTER (Language Teacher Emotion Regulation)

**Correlation is significant at the 0.01 level (2 tailed)

As reported in Table 2, the results of the Pearson product-moment correlation analysis revealed that Self-Expectation (*r* = 0.98, *p* < 0.01), Teachers’ Duties (*r* = 0.93, *p* < 0.01), External Influential Factors (*r* = 0.90, *p* < 0.01), Pedagogy (*r* = 0.89, *p* < 0.01), Instructional Skills and Knowledge (*r* = 0.97, *p* < 0.01), Teachers’ Citizenship Behavior (*r* = 0.89, *p* < 0.01), General Autonomy (*r* = 0.82, *p* < 0.01), Curriculum Autonomy (*r* = 0.81, *p* < 0.01), and Amotivation (*r* = -0.68, *p* < 0.01) are positively correlated with TI. Moreover, Extrinsic Material regulation (*r* = 0.79, *p* < 0.01), Extrinsic social regulation (*r* = 0.76, *p* < 0.01), Introjected regulation (*r* = 0.75, *p* < 0.01), Identified regulation (*r* = 0.74, *p* < 0.01), as well as Intrinsic motivation (*r* = 0.72, *p* < 0.01) are associated with TI.

Additionally, Self-Expectation (*r* = 0.55, *p* < 0.01), Teachers’ Duties (*r* = 0.49, *p* < 0.01), External Influential Factors (*r* = 0.47, *p* < 0.01), Pedagogy (*r* = 0.45, *p* < 0.01), Instructional Skills and Knowledge (*r* = 0.43, *p* < 0.01), Teachers’ Citizenship Behavior (*r* = 0.43, *p* < 0.01), General Autonomy (*r* = 0.54, *p* < 0.01), Curriculum Autonomy (*r* = 0.56, *p* < 0.01), Amotivation (*r* = -0.58, *p* < 0.01), Extrinsic Material regulation (*r* = 0.68, *p* < 0.01), Extrinsic Social regulation (*r* = 0.65, *p* < 0.01), Introjected regulation (*r* = 0.75, *p* < 0.01), Identified regulation (*r* = 0.74, *p* < 0.01), and Intrinsic motivation (*r* = 0.60, *p* < 0.01) were significantly correlated with TER.

**Table 3:** Model fit indices (Model 1)

Fitting indexes	$$ \chi 2$$	$$ df$$	$$ \chi 2/df$$	RMSEA	GFI	NFI	CFI
Cut value			<3	< 0.1	> 0.9	> 0.9	> 0.9
Model 1	889.48	317	2.80	0.06	0.92	0.94	0.95

*Note*: CFI (Comparative fit index); GFI (Goodness-of-fit index) RMSEA (Root mean square error of approximation); NFI (Normed fit index)

The results presented in Table 3 suggest that the model fit indices meet the required criteria for a good fit, as evidenced by the Chi-Square/df ratio (2.80), RMSEA (0.06), GFI (0.92), NFI (0.94), and CFI (0.95).

**Fig. 1:**
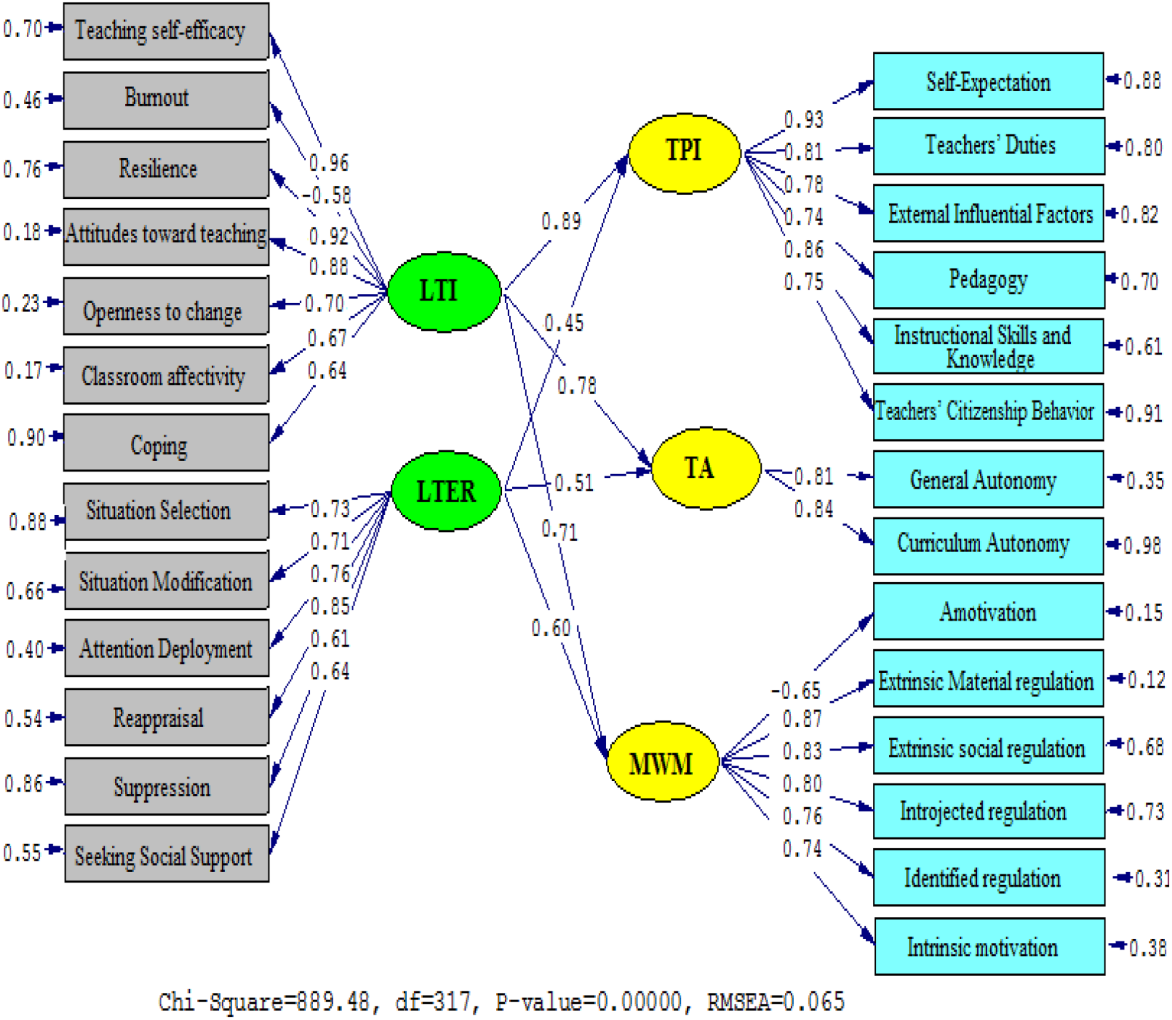
Diagrammatic representation of path coefficient values for the connections between TI, TER, TPI, TA, and TWM (Model 1). *Note*: LTI (Language Teacher Immunity); LTER (Language Teacher Emotion Regulation); TPI (Teacher Professional Identity); TA (Teacher Autonomy); MWM (Multidimensional Work Motivation)

**Fig. 2:**
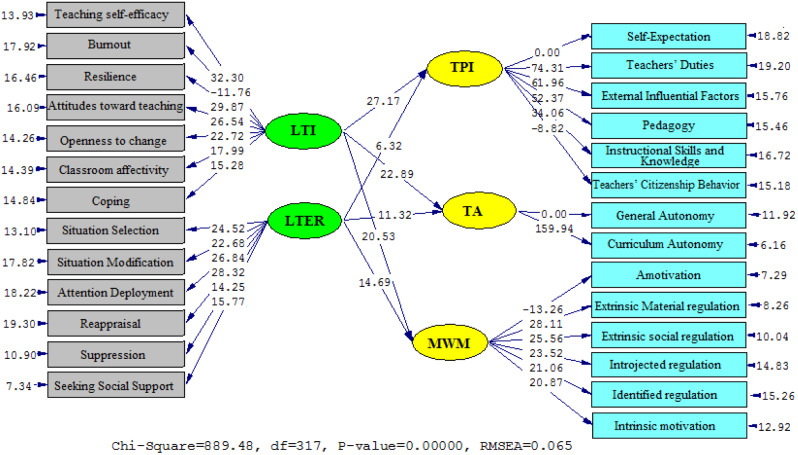
T Values to determine the significance of path coefficients (Model 1). *Note*: LTI (Language Teacher Immunity); LTER (Language Teacher Emotion Regulation); TPI (Teacher Professional Identity); TA (Teacher Autonomy) (MWM (Multidimensional Work Motivation)

Figures 1 and 2 from the study suggest an inverse relationship between TI, TER, TPI, TA, and TWM. The statistical data of Model 2 collected in Table 3 portray suitable data results in the form of RMSEA (0.067), GFI (0.914), NFI (0.925), and CFI (0.940), implying a satisfactory fit. Notably, the study found that there is an inverse correlation between TI and TPI (*β* = 0.89, *t* = 27.17), TI and TA (*β* = 0.78, *t* = 22.89), and TI and TWM (*β* = 0.71, *t* = 20.53), whereas positive correlations exist between LTER and TPI (*β* = 0.45, *t* = 6.32), TI and TA (*β* = 0.51, *t* = 11.32), and TI and TWM (*β* = 0.60, *t* = 14.69).

The outcomes extracted from Table 4 demonstrate that the goodness-of-fit indices belonging to Model 2 are appropriate. This is mainly indicated by the chi-square/df ratio (2.91), RMSEA (0.06), GFI (0.91), NFI (0.92), and CFI (0.94) measures, which reflect favorable values. The results are reported in Table 3.

**Table 4:** Model fit indices (Model 2)

Fitting indexes	$$ \chi 2$$	$$ df$$	$$ \chi 2/df$$	RMSEA	GFI	NFI	CFI
Cut value			<3	<0.1	> 0.9	> 0.9	> 0.9
Model 2	7157.64	2455	2.91	0.06	0.91	0.92	0.94

*Note*: CFI (Comparative fit index); GFI (Goodness-of-fit index) RMSEA (Root mean square error of approximation); NFI (Normed fit index)

**Fig. 3:**
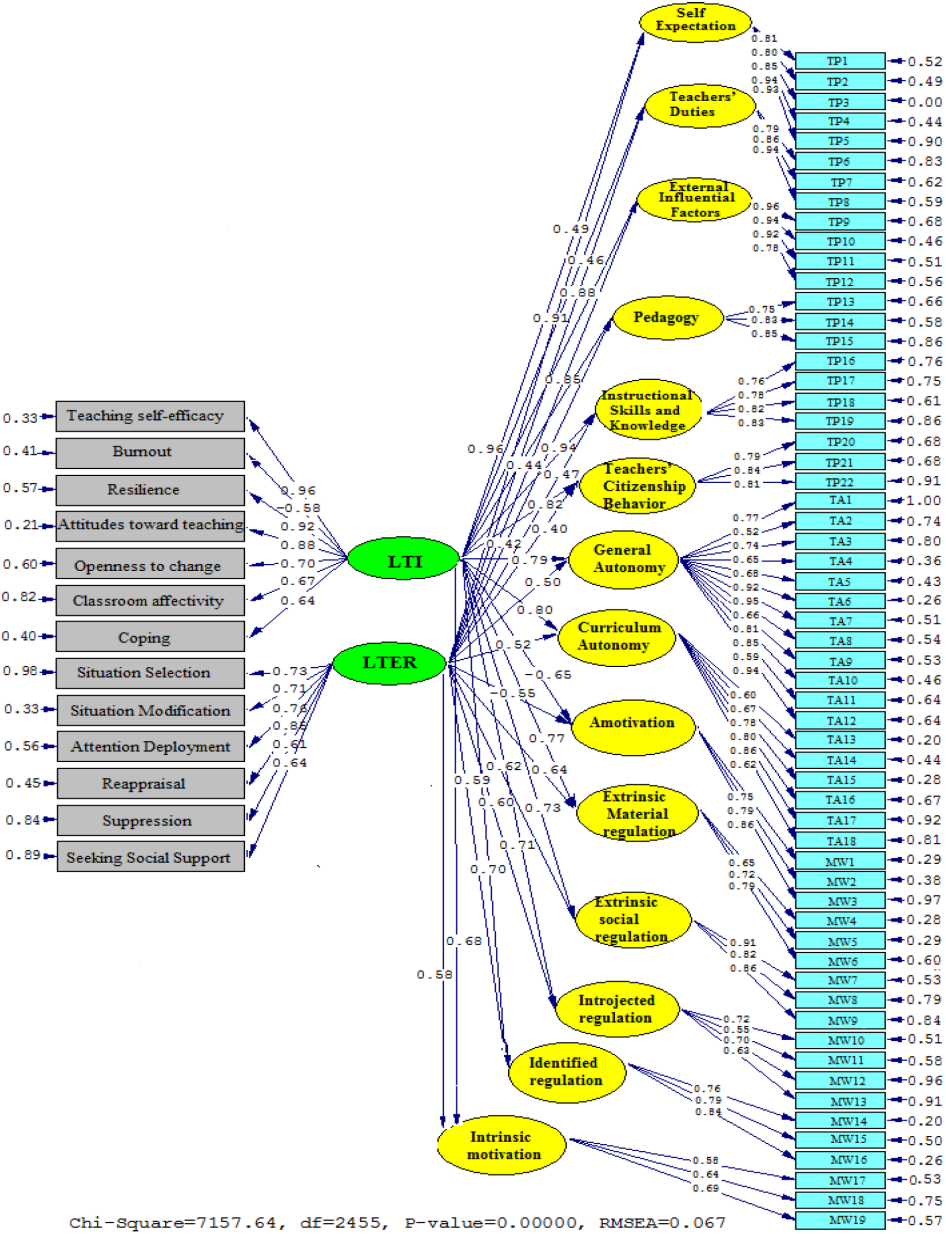
Diagrammatic representation of path coefficient values for the connections between TI, TER, TPI, TA, and TWM subscales (Model 2). *Note*: LTI (Language Teacher Immunity); LTER (Language Teacher Emotion Regulation)

In Fig. 3 as well as Supplementary Fig. 1 (refer to Additional File 1), a graphical representation of the correlation between TI, TER, TPI, TA, and TWM was shown. Investigating the correlation between TI and the other subscales, results found are Self-Expectation (*β* = 0.96, *t* = 35.82), Teachers’ Duties (*β* = 0.91, *t* = 32.26), External Influential Factors (*β* = 0.88, *t* = 30.64), Pedagogy (*β* = 0.85, *t* = 29.41), Instructional Skills and Knowledge (*β* = 0.94, *t* = 34.75), Teachers’ Citizenship Behavior (*β* = 0.82, *t* = 27.36), General Autonomy (*β* = 0.79, *t* = 25.75), Curriculum Autonomy (*β* = 0.80, *t* = 25.92), Amotivation (*β* = -0.65, *t* = -15.36), Extrinsic Material Regulation (*β* = 0.77, *t* = 24.81), Extrinsic Social Regulation (*β* = 0.73, *t* = 24.12), Introjected Regulation (*β* = 0.71, *t* = 23.90), Identified regulation (*β* = 0.70, *t* = 22.74), and Intrinsic Motivation (*β* = 0.68, *t* = 17.63). These results indicate that TI has a very strong positive relationship with all the subscales except Amotivation, which has a moderate negative relationship. This means that higher levels of TI are associated with higher levels of self-expectation, teachers’ duties, external influential factors, pedagogy, instructional skills and knowledge, teachers’ citizenship behavior, general autonomy, curriculum autonomy, extrinsic material regulation, extrinsic social regulation, introjected regulation, identified regulation, and intrinsic motivation, and lower levels of amotivation. Similarly, the findings of the relationship between TER and other subscales are Self-Expectation (*β* = 0.49, *t* = 8.21), Teachers’ Duties (*β* = 0.46, *t* = 7.86), External Influential Factors (*β* = 0.44, *t* = 7.31), Pedagogy (*β* = 0.42, *t* = 6.44), Instructional Skills and Knowledge (*β* = 0.47, *t* = 8.02, Teachers’ Citizenship Behavior (β = 0.40, *t* = 5.20), General Autonomy (*β* = 0.50, *t* = 10.75), Curriculum Autonomy (*β* = 0.52, *t* = 11.24), Amotivation (*β* = -0.55, *t* = -11.52), Extrinsic Material Regulation (*β* = 0.64, *t* = 14.82), Extrinsic Social Regulation (*β* = 0.62, *t* = 14.05), Introjected Regulation (*β* = 0.60, *t* = 13.64), Identified regulation (*β* = 0.59, *t* = 13.11), and Intrinsic Motivation (*β* = 0.58, *t* = 12.87). These results indicate that TER has a moderate to strong positive relationship with all the subscales except Amotivation, which has a strong negative relationship. This means that higher levels of TER are associated with higher levels of self-expectation, teachers’ duties, external influential factors, pedagogy, instructional skills and knowledge, teachers’ citizenship behavior, general autonomy, curriculum autonomy, extrinsic material regulation, extrinsic social regulation, introjected regulation, identified regulation, and intrinsic motivation, and lower levels of amotivation.

## Discussion

This study aimed to explore how EFL teachers’ TI and TER affect their TPI, TA, and TWM. The findings, especially Model 1, showed that TI and TER are significant predictors of TPI, TA, and TWM. This means that having a high level of protection and a strong ability to regulate emotions can foster self-determination, autonomy, enthusiasm, resilience, and persistence. Conversely, neglecting emotional balance and having maladaptive TI can be harmful. Thus, teachers should use more reflective and introspective strategies to deal with the complex challenges and changes in educational settings. It is also important to increase teachers’ understanding of the vital role of PI, TA, and TWM in their work performance and the underlying principles behind them.

The results of models 1 and 2 revealed that TI and TER significantly predicted TPI for the language teachers. PI is a complex and context-dependent construct, influenced by various factors. Sometimes, individuals may challenge their own or others’ identities [76]. Therefore, language teachers’ self-perception and interaction with others are crucial for their TPI development and (re)construction [18]. EFL teachers’ professional identities are socially constructed through dialogues and interactions with the self and other group members. This highlights the role of TI and TER in maintaining balance and facilitating the process of TPI change and growth for language teachers. This finding is also in line with [51] claim that a person’s TPI affects their work commitment and productivity. The findings suggest that EFL teachers with high levels of TI and effective TER are more likely to feel confident in their ability to handle the challenges of teaching, leading to higher levels of self-efficacy [9, 75]. This, in turn, may contribute to a stronger TPI and a sense of competence as educators. Additionally, EFL teachers who are able to regulate their emotions effectively and maintain high levels of TI are more likely to experience positive emotions, such as enjoyment and satisfaction, in their work. This can result in higher levels of job satisfaction, which is another important component of TPI [76]. Ultimately, EFL teachers who are satisfied with their jobs are more likely to view teaching as a meaningful and fulfilling career, further strengthening their TPI.

The results for the second research question showed that TI and TER can predict TA among the EFL teachers. According to Model 2, the EFL teachers’ high TI and emotional balance enhance their general and curricular TA, enabling them to deal with challenges and diversity in their profession with confidence and emotional engagement. As a result, teachers can take more responsibility, ensure efficiency, and exercise cognitive and affective control during instruction, leading to increased TA. This agrees with [77] finding of positive outcomes for teachers with high TA in their field. Alternatively, the results could also imply that the EFL teachers who have developed their professional identities and gained peer recognition are more able to pursue their professional and personal goals effectively and autonomously [78]. Based on the study’s findings, it is plausible to argue that EFL teachers with high levels of TI are more likely to experience TA in the classroom [8]. These teachers are better equipped to handle the demands of their job, including managing classroom behavior, dealing with difficult students, and meeting deadlines. Consequently, they feel empowered to make decisions about their teaching practices and exercise their TA in the classroom. Additionally, EFL teachers who can regulate their emotions effectively are better equipped to handle the challenges of teaching, such as managing classroom conflicts and difficult students [21]. This ability leads to positive emotions, such as enjoyment and satisfaction, which further enhance their sense of TA in the classroom [14]. Conversely, teachers who struggle with TER may experience negative emotions, such as frustration and anxiety, which can diminish their sense of TA and lead to a sense of powerlessness in the classroom.

The third research question examined how EFL teachers’ TI and TER affect their TWM. The results showed that TI and TER significantly predicted EFL teachers’ TWM. That is, the study revealed that the EFL teachers who engage in reflective and evaluative processes improved their TWM. According to Model 2, TI and TER negatively influenced amotivation, one of the sub-components of work motivation. Therefore, it is likely that TI and TER reduced the reluctance of the EFL teachers to engage in various educational activities by enabling them to overcome obstacles through continuous monitoring, planning, and assessment. Additionally, Model 2 indicated that the reflective practices of EFL teachers positively influenced intrinsic motivation, identified regulation, introjected regulation, and extrinsic regulation. Similar findings were reported by [13], who found that TER enhances the engagement and motivation of EFL instructors. The results of this study are also consistent with those of [50], who reported that the development of TPI among teachers leads to improved work effectiveness. Besides, the current study agrees with the research by [45], which established a positive correlation between TER and teacher self-efficacy and self-reflection. Likewise, the outcomes of this study are in line with those of [13], who demonstrated that ER had a significant predictive capacity for both self-efficacy and job satisfaction. Moreover, [8] found that TER was associated with reflective teaching and job commitment in the Iranian tertiary context, which is consistent with the results of this research. Based on the findings of the study, it may be argued that the EFL teachers who have a high level of TI are less likely to experience burnout and stress, which can negatively impact their motivation to work. That is, the EFL teachers with high TI are better equipped to handle the demands of their job, such as managing classroom behavior, dealing with difficult students, and meeting deadlines. As a result, they are more likely to feel motivated to continue teaching and to perform well in their job. Additionally, along with the findings, it may be stated that the EFL teachers who are able to regulate their emotions effectively are better able to handle the challenges of teaching, such as dealing with difficult students or managing classroom conflicts [11]. They are also more likely to experience positive emotions, such as enjoyment and satisfaction, which can enhance their motivation to work [13]. In contrast, teachers who struggle with emotion regulation may experience negative emotions, such as frustration and anxiety, which can lead to burnout and reduced motivation to work.

### Conclusions and pedagogical implications

The benefits of TI and TER for language teachers are well-established, but their relationship with other important constructs such as TPI, TA, and TWM is still unclear. This study revealed that TI and TER had a significant positive impact on TPI, TA, and TWM. Moreover, the study provided strong empirical evidence that by developing TI and TER, teachers can improve their teaching practices in the face of instructional challenges and uncertainties. These findings offer a more optimistic outlook on the teaching profession, increasing the chances of success rather than failure.

As this study shows that TI and TER are essential factors that enhance TPI, TA, and TWM, these findings have implications for various stakeholders in the field of language instruction, such as language teacher educators, language teachers, government agencies, decision-makers, school administrators, and language learners. First, for language teacher educators, these findings can help design more effective pre-service and in-service programs that foster TI and TER among language teachers. For example, they can incorporate activities that help teachers explore their identity and emotion as language professionals, such as narrative writing, reflective journals, peer coaching, and mentoring. They can also provide feedback and support that can enhance teachers’ self-efficacy, self-regulation, and motivation, which are key components of TI and TER. Second, for language teachers, these findings suggest that they should adopt a reflective approach to language teaching, which can help them become aware of the strengths and weaknesses of their teaching and learning practices. For example, they can use self-assessment tools, such as the ones used in this study, to evaluate their TPI, TA, and TWM, and identify areas for improvement. They can also engage in collaborative reflection with their colleagues, such as through professional learning communities, action research, or lesson study, to share their experiences and insights, and learn from each other. Third, for government agencies and decision-makers, these findings can inform their policies and actions that are responsible for the quality and outcomes of language instruction programs. For example, they can allocate more resources and funding for teacher education programs that focus on developing TI and TER among language teachers. They can also establish standards and guidelines that can help teachers and schools implement effective practices that can enhance TPI, TA, and TWM. They can also monitor and evaluate the impact of these policies and actions on the teachers and learners’ performance and satisfaction. Fourth, for school administrators, these findings can be especially useful for improving TPI, TA, and TWM among their teachers. For example, they can create a supportive and empowering school culture that values and respects teachers’ identity and emotion, and encourages their professional growth and autonomy. They can also provide opportunities and incentives for teachers to participate in continuous learning and development activities, such as workshops, conferences, or online courses, that can help them improve their TI and TER. They can also recognize and reward teachers who demonstrate high levels of TPI, TA, and TWM, and inspire others to do the same. Last but not least, for language learners, these findings can have positive effects on their learning outcomes and experiences. For example, they can benefit from having teachers who have a clear and strong sense of who they are and how they feel as language professionals, and who can adapt their teaching methods and strategies to suit the learners’ needs and preferences. They can also enjoy more autonomy and well-being in their learning process, as they can have more choices and control over their learning goals, activities, and assessment, and receive more support and feedback from their teachers and peers.

This study has some limitations that should be considered when interpreting the results and that suggest directions for future research. First, this study did not use a qualitative or data-driven approach to explore the perspectives of teachers and educators on the constructs of interest. Therefore, future research could use more mixed-method approaches to examine the association between TI, TER, TPI, TA, and amotivation in a more comprehensive way. Second, this study did not consider demographic factors that may affect TI, such as their cultural and socioeconomic background, their area of expertise, their level of proficiency, or their pedagogical training. Future studies could investigate how these factors influence TI, reflective teaching, and work motivation. Third, the results of this study, like any other academic research, need to be replicated in other EFL contexts to provide more evidence for educators, trainers, and practitioners. Finally, to fully understand the relationship between TI, TER, TPI, TA, and amotivation among EFL teachers, a longitudinal study is needed. Such a study would reveal how these variables change over time. As this study was cross-sectional, a longitudinal study is essential to capture the dynamics of these variables.

### Electronic supplementary material

Below is the link to the electronic supplementary material.

Supplementary Material 1: T Values to Determine the Significance of Path Coefficients (Model 2)

## Data Availability

The dataset of the present study is available upon request from the corresponding author.
