# Supplementary material for: A voyage of discovering the impacts of teacher immunity and emotion regulation on professional identity, autonomy, and work motivation in Iranian EFL landscape
Source: BMC Psychol. 2024 Jan 22;12:43. doi: 10.1186/s40359-024-01544-9 (PMC10801986; doi:10.1186/s40359-024-01544-9)
Supplement: Supplementary file 1 — Supplementary Material 1: T Values to Determine the Significance of Path Coefficients (Model 2) [file 40359_2024_1544_MOESM1_ESM.pdf]

Supplementary Figure 1: *T Values to Determine the Significance of Path Coefficients (Model 2)*

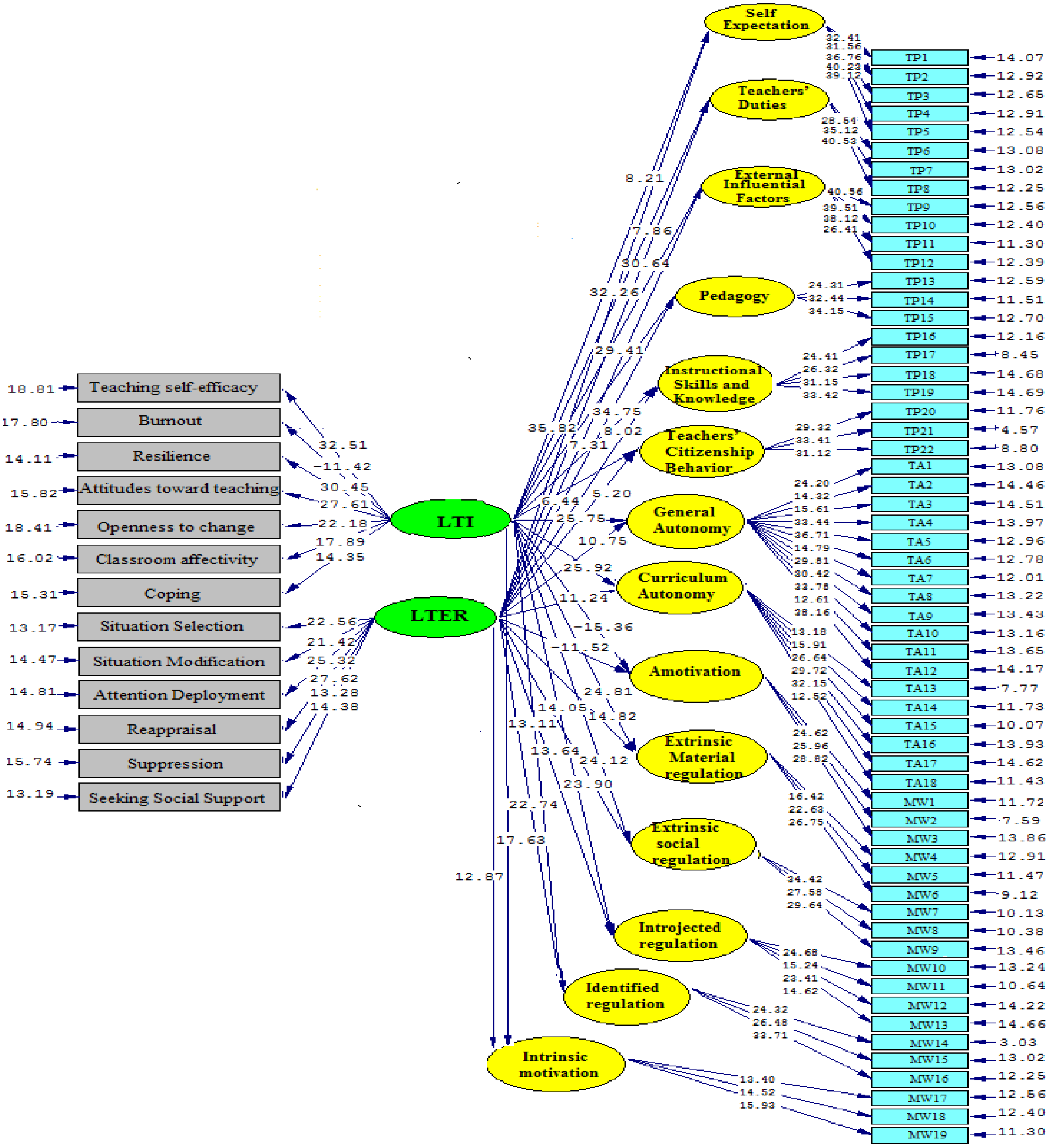

Chi-Square=7157.64, df=2455, P-value=0.00000, RMSEA=0.067

NOTE: LTI (Language Teacher Immunity); LTER (Language Teacher Emotion Regulation)
